# Diagnosis methods for pancreatic cancer with the technique of deep learning: a review and a meta-analysis

**DOI:** 10.3389/fonc.2025.1597969

**Published:** 2025-08-20

**Authors:** Yuanbo Bi, Dongrui Li, Ruochen Pang, Chengxv Du, Da Li, Xiaoyv Zhao, Haitao Lv

**Affiliations:** ^1^ Department of Hepatobiliary Surgery, The Second Hospital of Hebei Medical University, Shijiazhuang, Hebei, China; ^2^ Department of Spine Surgery, The Third Hospital of Hebei Medical University, Shijiazhuang, Hebei, China; ^3^ Department of Oncology, Hebei Medical University, Shijiazhuang, Hebei, China

**Keywords:** pancreatic cancer (PC), deep learning, diagnosis methods, research trends, meta-analysis

## Abstract

**Background:**

Early diagnosis can significantly improve survival rate of Pancreatic ductal adenocarcinoma (PDAC), but due to the insidious and non-specific early symptoms, most patients are not suitable for surgery when diagnosed. Traditional imaging techniques and an increasing number of non-imaging diagnostic methods have been used for the early diagnosis of pancreatic cancer (PC) through deep learning (DL).

**Objective:**

This review summarizes diagnosis methods for pancreatic cancer with the technique of deep learning and looks forward to the future development directions of deep learning for early diagnosis of pancreatic cancer.

**Methods:**

This study follows the PRISMA-ScR (Preferred Reporting Items for Systematic Reviews and Meta-Analyses Extension for Scoping Reviews) guidelines, retrieving studies on deep learning for early pancreatic cancer diagnosis from PubMed, Embase, Web of Science, IEEE, and Cochrane Library over the past 5 years. Inclusion criteria were studies involving PDAC patients, using deep learning algorithms for diagnosis evaluation, using histopathological results as the reference standard, and having sufficient data. Two reviewers independently screened and extracted data. Quality was assessed using QUADAS-2, with StataMP 17 for meta-analysis.

**Results:**

In this study, 422 articles were retrieved, and 7 were finally included for meta-analysis. The analysis showed that the accuracy of deep learning in the early diagnosis of pancreatic cancer was 80%-98.9%, and the combined sensitivity, specificity and AUC were 0.92 (95% CI: 0.85-0.96), 0.92 (95% CI: 0.85-0.96), and 0.97 (95% CI: 0.95-0.98). The positive and negative likelihood ratio were 11.52 (95% CI, 6.15-21.55) and 0.09 (95% CI, 0.04-0.17). Endoscopic ultrasound (EUS) and Contrast-Enhanced Computed Tomography (CE-CT) were the main diagnostic methods. Non-imaging diagnostic methods such as deep learning urine markers, disease trajectory also performed good diagnostic potential.

**Conclusions:**

Artificial intelligence (AI) technology holds promise for clinical guidance in pancreatic cancer risk prediction and diagnosis. Future research may focus on leveraging diverse data sources like genomics and biomarkers through deep learning; utilizing multi - center or international samples; tackling the challenge of early diagnosis for small pancreatic cancers; enhancing the explainability of AI models and multi-modal approaches.

## Introduction

### Background

Pancreatic Ductal Adenocarcinoma (PDAC) is a highly aggressive and lethal malignant tumor of the pancreas. Currently, it is the fourth leading cause of cancer death and is projected to become the second by 2030 (1, 2). The high mortality rate of PDAC is mainly due to the challenge of its diagnosis (3). Early diagnosis can significantly improve the 5-year survival rate (4). But due to the similar attenuation of early PDAC to healthy pancreas, approximately 40% of pancreatic tumors less than 2cm are missed on abdominal computed tomography (CT) (5–8). However, PDAC is asymptomatic until it progresses to an advanced stage. At the time of diagnosis, only about 20% of cases are suitable for surgical resection (9).

It is clear that if cancer can be detected early and appropriate surgical and systemic treatments provided, the survival rate of PC could be significantly improved (10). Imaging techniques play a crucial role in the diagnosis of PDAC. Current clinical imaging modalities include endoscopic ultrasound (EUS), CT, and positron emission tomography-computed tomography (PET/CT), each with its own advantages and disadvantages in clinical application (11–13). In the traditional process of medical image analysis, experienced radiologists are required. With artificial intelligence technology, radiologists can be liberated from tedious and repetitive tasks to handle those that require more creativity. The gradual development of deep learning and radiomics provides a new perspective for the use of the above as the diagnosis and screening of early PC (14).

Deep learning (DL) has recently made progress on many problems, especially in medical imaging diagnosis, and in some cases has even surpassed human performance (13). Deep learning constitutes a specialized branch of machine learning that employs multi-layered (deep) neural networks to automatically extract hierarchical features from high-dimensional data, yielding breakthrough performance in tasks such as image recognition, speech processing, and natural language understanding. Machine learning (ML), in turn, is a sub-discipline of AI; it leverages statistical techniques to enable computer systems to learn patterns from data and make predictions or decisions without explicitly programming every rule (6, 7). AI is the broad discipline devoted to enabling machines to exhibit intelligent behavior. Its canonical formulation, first articulated by John McCarthy, defines AI as “the science and engineering of making intelligent machines, especially intelligent computer programs that perform tasks which, if carried out by humans, would require human intelligence” (15). Consequently, AI, ML, and DL exhibit a nested relationship: AI ⊃ ML ⊃ DL. In recent years, a large number of studies related to deep learning have been published, but some of them seem to blur the definitions of deep learning and machine learning. Deep learning is a subset of ML algorithms. The conceptual difference between the two is easy to overlook (16).

With the increase in research, various new diagnostic markers for PC diagnosis using deep learning have emerged one after another (17). Based on the aforementioned, this study aims to assess the clinical relevance and translational potential of deep learning algorithms in the early detection of pancreatic cancer. We systematically review applications across imaging modalities, urinary biomarkers, and disease-trajectory models, with particular emphasis on diagnostic methods, neural-network architectures, and downstream clinical utility. In addition, we performed a comprehensive meta-analysis of the diagnostic accuracy reported by the selected studies.

## Methods

### Overview

In this review, PRISMA-ScR (Preferred Reporting Items for Systematic Reviews and Meta-Analyses Extension for Scoping Reviews) guidelines were followed to ensure the transparency and reliability of this study.

### Search strategy

#### Search sources

**Figure 1:**
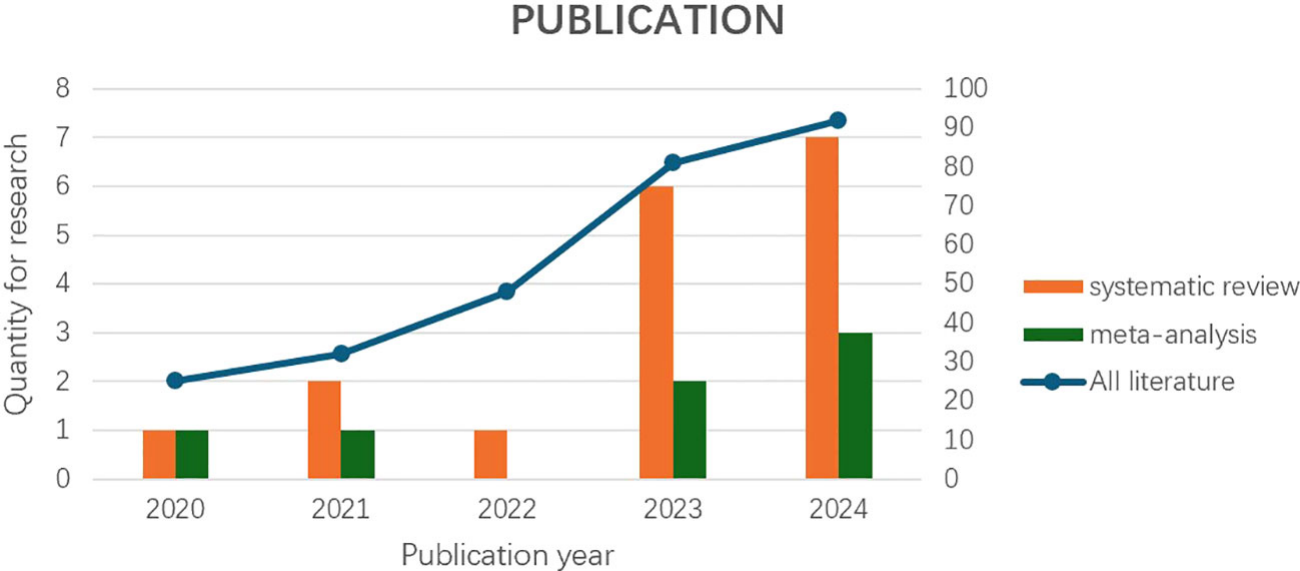
“Systematic review and meta-analysis“ based on the axis labels on the left, and “All literature” based on the axis labels on the right (classified by year).

We searched five databases (PubMed, Embase, Web of science (WOS), IEEE, and Cochrane Library) for studies on DL for early diagnosis of PDAC published between December 31, 2019 and December 31, 2024 (**Figure 1**).

#### Search terms

To develop the search strategy, 2 experts in the fields of artificial intelligence and hepatobiliary surgery were consulted, and previous relevant reviews were consulted to identify subject headings and free words. Search terms were selected according to the target intervention (i.e., deep learning), the target disease (i.e., PC), and the purpose of the intervention (i.e., early diagnosis). Details of the exact search terms used to search each database are provided in **Supplementary File 1**.

### Study eligibility criteria

Following the PICOS principle, studies meeting the following inclusion criteria were admitted: (1) included patients with a diagnosis of PDAC; (2) develop or use clinical predictive models or deep learning algorithms to evaluate PC diagnosis instead of segmentation; (3) used histopathologic results as the reference standard (4) had sufficient data to reconstruct the 2 × 2 contingency table to be included.

The following studies were excluded: (1) reviews, case reports, letters, commentaries, errata, meta-analyses and studies published only as conference abstracts; (2) Studies were also excluded if they lacked internal or external validation; (3) The number of the same diagnostic method was less than 3; (4) The sample size was less than 50; (5) The sample was not on a per-patient basis.

### Study selection

The eligibility of articles was determined by two reviewers (Yuanbo Bi and Dongrui Li), who independently screened the titles and abstracts of the search results. In case of disagreement, the third reviewer (Ruochen Pang) will intervene and decide together. To measure inter-reviewer agreement, we calculated Cohen kappa, which was 0.892 for title and abstract screening and 0.810 for full-text screening, with almost perfect agreement (13, 18).

### Data extraction

To accurately extract data from the included studies from the selected studies, a data extraction table was created using Microsoft Excel, and the data extraction fields are described in **Supplementary File 3** and filled in using the included studies. Two reviewers independently performed the process, and any disagreements between the two reviewers were resolved through discussion by the intervention of a third reviewer.

### Data synthesis

To estimate the accuracy of deep learning algorithms, we conducted a meta-analysis of studies that provided sufficient data to construct contingency tables. StataMP 17 (64-bit) was used for forest plot, funnel plot, SROC curve, heterogeneity test and meta-analysis. Studies evaluating deep learning in robotic programs were not included in the analysis. Microsoft Excel was used to manage the comprehensive data. Endnote X9 was used for literature management.

### Quality assessment

The methodological quality of eligible articles was determined by two authors (Yuanbo Bi and Dongrui Li) using the QUADAS-2. QUADAS-2 tool is recommended for use in systematic reviews of diagnostic accuracy based on sources of bias and variation and comprises four domains: patient selection, index test, reference standard, and flow and timing. Each domain is assessed in terms of risk of bias, and the first three domains are also assessed in terms of concerns regarding applicability. Each component contains several questions used to help judge the risk of bias (low, high or unclear) (13).

## Results

### Search results

**Figure 2:**
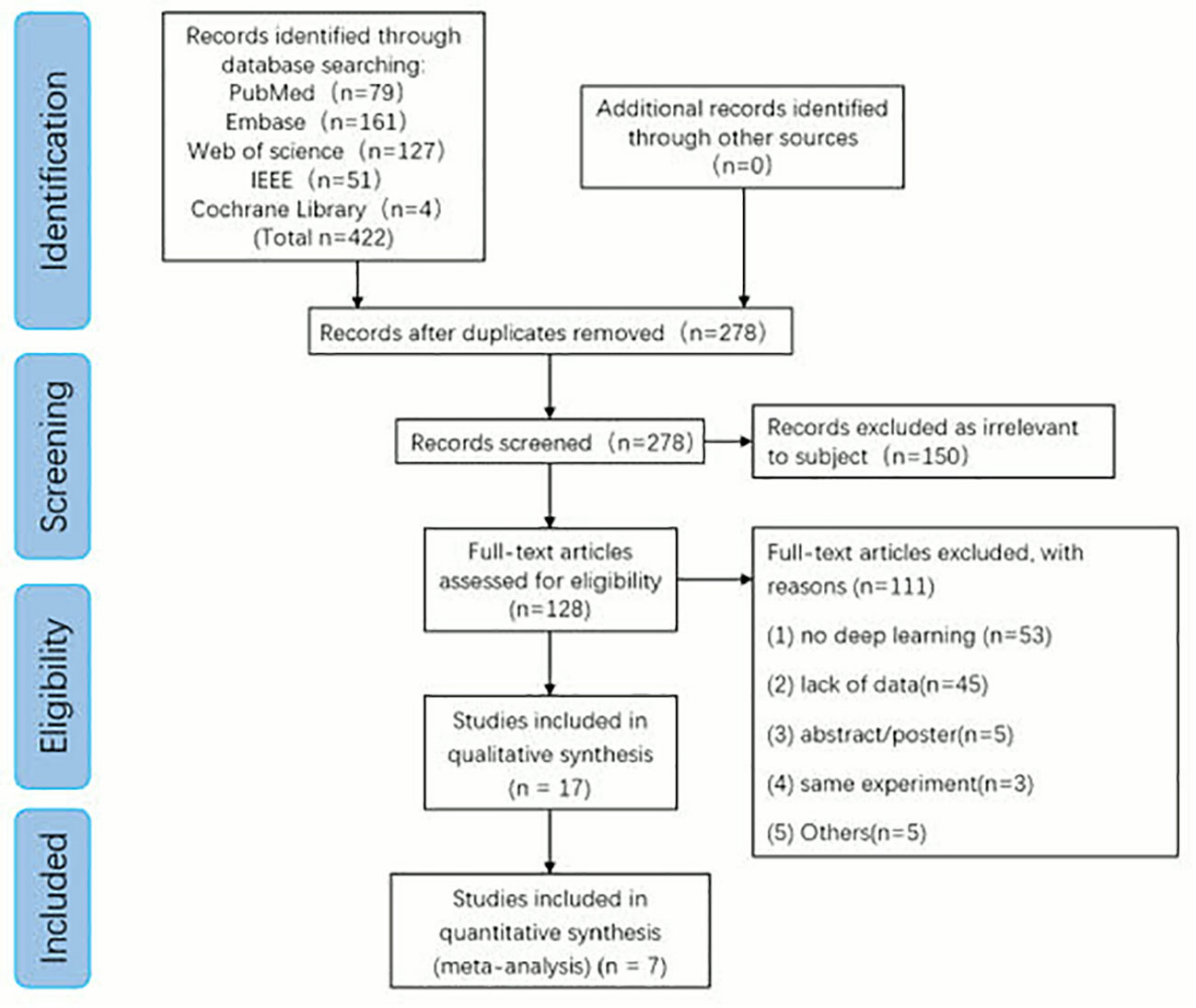
Preferred reporting items for systematic review and meta-analysis of diagnostic test accuracy studies (PRISMA) flow diagram for study selection.

We initially identified 422 articles using five publicly available online databases: PubMed (n=79), Embase (n=161), Web of science (n=127), IEEE (n=51), Cochrane Library (n=4) +Additional records identified through other sources (n=0) entered into PRISMA-ScR flow chart. We excluded 144 replicates. Among the remaining studies, 150 articles unrelated to the research topic were excluded according to the abstract, and 17 articles were included in the systematic review after the full-text evaluation of the remaining 128 articles. A total of 7 articles met our inclusion criteria and were included in the meta-analysis (**Figure 2**).

### Study selection and characteristics

The results of 17 studies (out of 278) that met the inclusion criteria are summarized in **Supplementary File 2**. These studies were published between 2019 and 2024. The techniques applied included CE-CT (23.53%), EUS (29.41%), with one study using Contrast-enhanced harmonic endoscopic ultrasound (CH-EUS), pathological examination (11.76%), disease trajectories (11.76%), urine biomarkers (11.76%), RNA (5.88%), and DNA (5.88%). Among them, 7 studies met both the inclusion and exclusion criteria and were included in the meta-analysis (**Table 1**).

**Table 1:** Publications reporting on deep learning use in early diagnosis of pancreatic cancer.

No.	Paper	Year	Applications	Model	Sample		
					Total	PDAC	Non-PDAC
1	Chen et al. (5)	2023	CE-CT	CNN	256	109	147
2	Gu et al. (3)	2024	EUS	deep‐learning radiomics(DLR)	123	71	52
3	Kuwahara, T.et al. (6)	2023	EUS	Deep convolutional generative adversarial network (DCGAN)	161	117	44
4a	Liu et al. (20)	2020	CE-CT	CNN	189	101	88
4b	Liu et al. (20)	2020	CE-CT	CNN	363	281	82
5	Mandal et al. (14)	2024	CE-CT	nnU-Net + MIL +C NN	1577	179	1398
6	Naito et al. (27)	2021	EUS	CNN	120	81	39
7	Xiang et al. (21)	2024	CE-CT	ResNet50 network	84	33	51

### Systemic review

In this section, we systematically review the 17 included studies according to their diagnostic modalities, critically appraise the advantages and limitations of each method, and synthesize the corresponding findings.

#### CE-CT

In recent years, research on the early diagnosis of PC using deep learning-based CE-CT has gained significant attention. CE-CT remains a primary imaging modality for detecting (PC). However, the complex anatomical structure of the pancreas and the generally low contrast of these images present substantial challenges for accurate segmentation of pancreatic CT images. Precise pancreatic segmentation is critical in clinical practice, particularly for diagnosing and treating PC (19). Notably, CE-CT exhibits low sensitivity for small tumors, with approximately 40% of tumors smaller than 2 cm being undetected (5, 20).

The accuracy of deep learning-based CE-CT for the early diagnosis of PC ranges from 80% to 98.90%, with sensitivity ranging from 0.79 to 0.973, specificity from 0.76 to 1, and AUC from 0.85 to 0.999. One study had a sample size of less than 100, resulting in statistical results that deviated significantly from those of the other three studies. Nevertheless, due to its minimal impact on the overall findings during subsequent subgroup analysis, this study was retained (21).

Chen et al. reported a sensitivity of 74.7% (95% CI: 64.5, 83.3) for malignant tumors smaller than 2 cm (5). Liu et al. provided sensitivities of 92.1%, 92.1%, and 63.1% for malignant tumors smaller than 2 cm in two internal validations and one external validation, respectively (20). The early diagnosis of PC smaller than 2 cm remains unclear, necessitating further research to confirm these findings, which may represent a promising new research direction.

#### EUS

Endoscopic ultrasound (EUS) is a non-invasive and highly precise technique for detecting pancreatic diseases and has become a widely used tool for diagnosing digestive system disorders (22–24). EUS-guided fine-needle aspiration biopsy (EUS-FNA/B) is considered the first-line method for the pathological diagnosis of PC due to its high accuracy (25). Reports indicate that the diagnostic sensitivity of EUS-FNA/B for pancreatic ductal adenocarcinoma (PDAC) is 85%–92%, with specificity ranging from 96% to 98%. Furthermore, it has been proven to be a feasible and safe technique with a complication rate of less than 1% (26).

In this study, the number of studies utilizing deep learning-based EUS for the early diagnosis of PC was the largest (n = 5), with accuracy ranging from 86.18% to 94.17%, sensitivity from 0.831 to 0.94, specificity from 0.822 to 1, and AUC from 0.9221 to 0.96 (3, 4, 6, 25, 27).

Among these, a prospective study on the early diagnosis of PC using deep learning CH-EUS demonstrated that the accuracy of CH-EUS NASTER-guided EUS-FNA was 93.8%, compared to 91.3% in the control group. Although the diagnostic rate of the CH-EUS MASTER group appeared higher, no statistically significant difference was observed between the two groups (p > 0.05). The AUC values for the CH-EUS MASTER group and the control group were 0.955 and 0.933, respectively (25). CH-EUS MASTER may serve as a promising real-time objective system for differentiating benign and malignant pancreatic masses. However, due to the small sample size (n = 39) and partial data loss, it was excluded from the subsequent meta-analysis.

Based on interval estimation, deep learning-based EUS image data appears slightly superior to CE-CT in terms of accuracy, sensitivity, specificity, and AUC, demonstrating greater stability. Nevertheless, additional studies are required to confirm these findings.

#### PET/CT

Although PET/CT holds significant value for the early diagnosis and differentiation of tumors, as well as for determining metastasis, its high cost and prolonged exposure time limit its application in the early diagnosis and screening of PC at present. Most current studies on deep learning-based PET/CT focus on tumor segmentation (28–30), rather than diagnosis. Consequently, no PET/CT studies were included in the meta-analysis.

#### Pathological examination

Deep learning of PC pathological tissues may have far-reaching significance for improving the accuracy of pancreatic cancer pathological diagnosis and targeted therapy (31). In routine pathological smears, stromal components account for 90% of the specimen and interact dynamically with the tumor, which poses a clinical challenge (32, 33). By leveraging deep learning and image analysis, information clues from stromal interactions can be extracted for the identification of new cancer biomarkers. This approach holds potential for enhancing diagnostic accuracy and providing deeper insights into the biology of pancreatic cancer (34).

Pancreatic cancer cells typically accumulate a large number of lipid droplets (LDs), which regulate lipid storage (35). To facilitate rapid diagnosis, Hong et al. proposes a deep convolutional neural network based automatic identification system for pancreatic cancer cells, which uses optical diffraction tomography to quantitatively image LDs of unstained cytology samples (35). The accuracy rate is 97.06% (± 1.021), and the AUC is 99.8%. This reflects the application prospects of deep learning in quantifying cell lipid droplet content in pancreatic cancer pathological examinations.

Fu et al. developed a deep learning model for the classification of pancreatic cancer pathological tissues. The automatic patch-level approach achieved a classification accuracy of 95.3%, and the whole-slide images-level (WSIs-level) approach achieved 100% (36). Fassler et al. quantitatively assessed T cells in the tumor microenvironment (TME) from multiplex immunohistochemistry (mIHC) WSIs of pancreatic ductal adenocarcinoma using the ColorAE: U-Net deep learning tool suite. They analyzed the spatial distribution of (CD3, CD4, CD8), B cells (CD20), macrophages (CD16), and tumor cells (K17) (33). This might promote the wider adoption of mIHC to support precision medicine, especially in the field of immunotherapy for pancreatic cancer.

#### Urine biomarkers

A study proposed the utilization of four urinary proteome biomarkers (creatinine, LYVE1, REG1B, and TFF1) to successfully develop a novel and highly efficient 1D CNN-LSTM model for the early diagnosis of PDAC. The proposed model is composed of one-dimensional convolutional neural networks (1D-CNNs) and long short-term memory (LSTM). Successful experiments and evaluations were conducted on 590 urine samples from the public dataset (183 healthy pancreatic samples, 208 samples with benign hepatobiliary diseases, and 199 PDAC samples). The study demonstrated that the 1-D CNN + LSTM model achieved an accuracy of 97% and an area under the curve (AUC) of 98% (37). This research shows a high accuracy and AUC, highlighting the great potential of urine markers for the early diagnosis of pancreatic cancer.

Linh, et al. introduce a label-free surface-enhanced Raman scattering sensor based on a three-dimensional plasmonic coral nanoarchitecture (3D-PCN), and successfully distinguished prostate cancer from pancreatic cancer by identifying urine with the aid of deep learning (38). However, there are few studies on the early diagnosis of pancreatic cancer using urine markers with deep learning, and more related research could be conducted to supplement this area. Acer et al. utilized non-invasive urine biomarkers and CA19-9, combined with seven machine learning models, to conduct early detection of PDAC in data from 590 participants, and found that the ensemble learning model performed best, with the GBC model achieving an accuracy of 92.99% (AUC = 0.9761) (39).

#### Disease trajectories

Deep learning models based on disease trajectories are used to integrate longitudinal clinical data from electronic medical records to infer the risk of PC, which may also be a breakthrough for the early diagnosis of pancreatic cancer (40).

Placido et al. predicted PC through deep learning of large sample disease trajectories, using (Electronic Health Records)EHR disease codes, and the best (Danish National Patient Registry) DNPR model’s performance was an area under the receiver operating characteristic (AUROC) curve of 0.88 (41). Park et al. used deep learning based on time series laboratory test results from EHR for the early detection of pancreatic cancer. The deep learning model demonstrated better performance on early detection (AUROC 0.671, CI 95% 0.667 – 0.675, p < 0.001) at 12 months prior to diagnosis compared to a logistic regression, xgboost, and a feedforward neural network baseline (42).

The predictive performance at the level demonstrated by deep-learning Disease trajectories may be useful for the initial design of real-world clinical predictive surveillance programs. This approach potentially provides a scalable workflow for community-level early cancer detection, shifting the focus from late-stage treatment to early-stage intervention.

#### RNA

Hong et al. proposed a novel deep learning framework, named HATZFS, for identifying driver biomarkers of pancreatic cancer using RNA differential expression data. This model integrates HDQN, GAT, and ZFS to construct a pancreatic cancer RNA transcriptional regulatory network involving lncRNA, miRNA, and mRNA relationships. To validate the effectiveness of this model, comprehensive experiments were conducted on a benchmark dataset containing 14 databases and compared with eight other algorithms (43). Overall, HATZFS not only provides the importance of all RNA molecules in the pancreatic cancer RNA regulatory network but also determines the set of driver nodes in this network as driver biomarkers.

#### DNA

Tumor type guides clinical treatment decisions for cancer. Histological diagnosis remains challenging, and genomic alterations have high diagnostic value for tumor types (36, 44).

Darmofal et al. developed Genome-Derived-Diagnosis Ensemble (GDD-ENS), a hyperparameter ensemble using deep neural networks to classify tumor types, based on genomic features from a dataset of 39,787 solid tumors sequenced with a clinical targeted cancer gene panel. GDD-ENS achieved an accuracy of 93% in high-confidence predictions for 38 cancer types (45).

### Methodological quality

**Figure 3:**
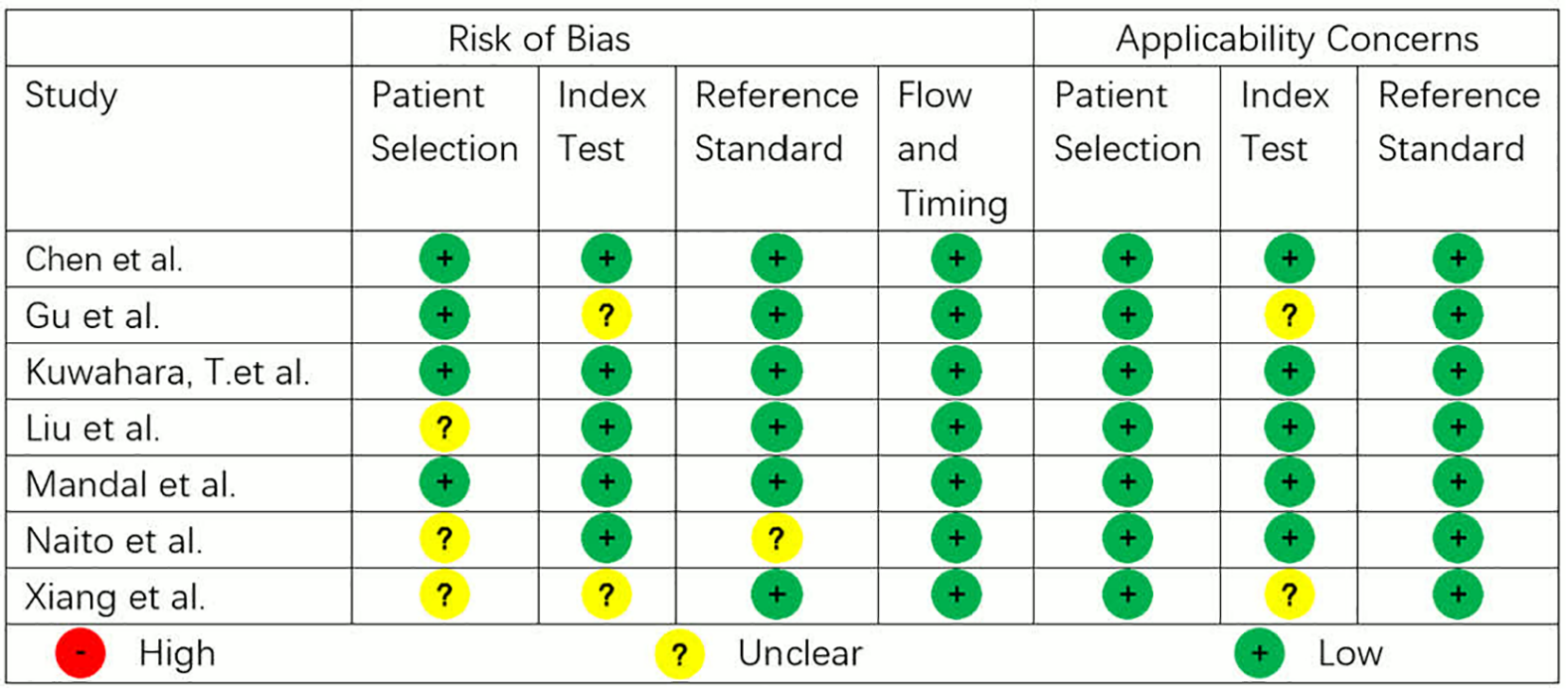
QUADAS-2 assessment of the studies.

The methodological quality of the studies according to QUADAS-2 assessment is illustrated in **Figure 3**. Three studies showed low risk of bias and applicability issues in all domains, while others showed unclear risks in some domains. No study had high risk of bias or applicability issues.

### Meta−analysis results

**Figure 4:**
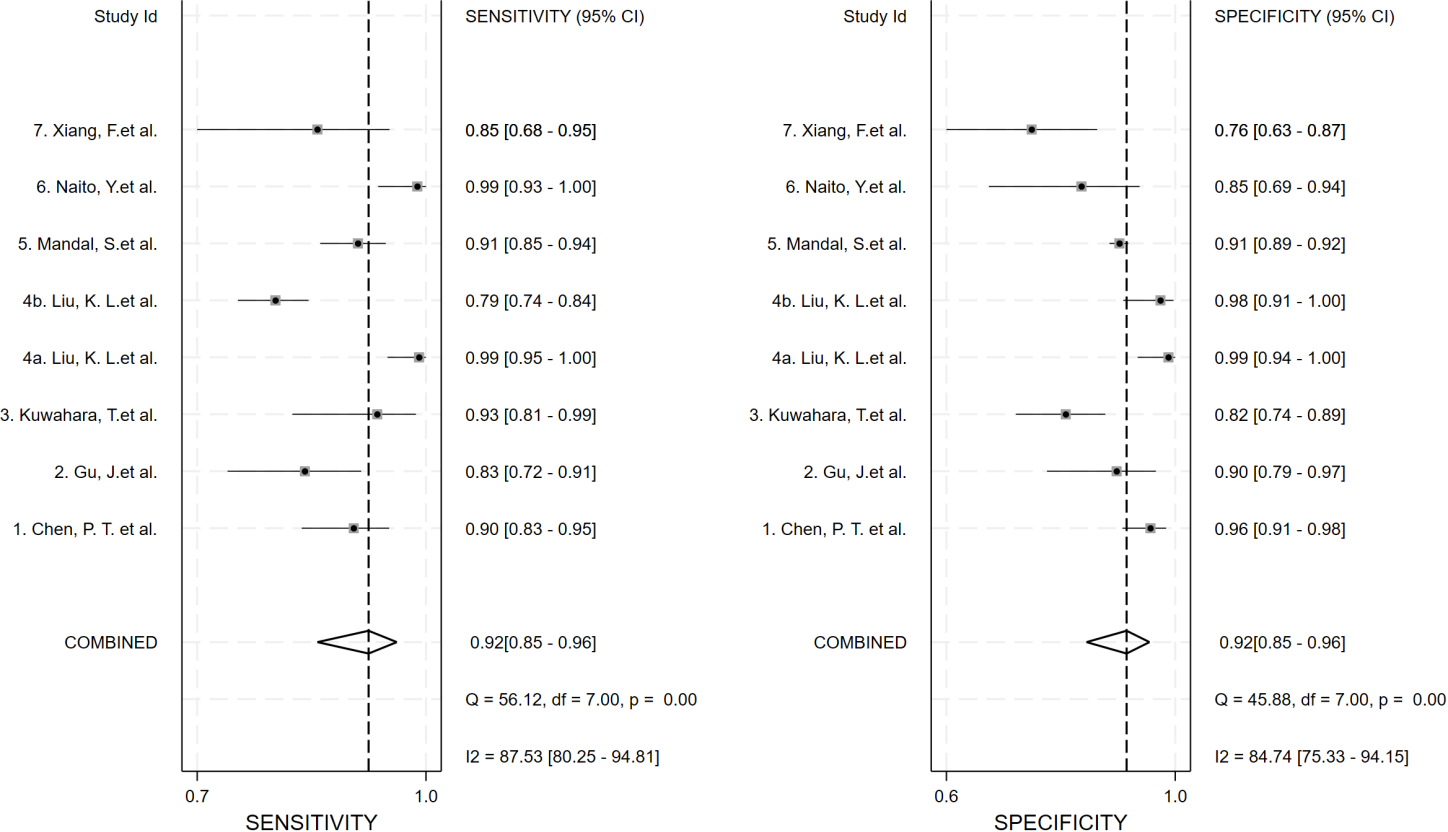
Forest plot of sensitivity and specificity of deep learning (DL) in identifying pancreatic tumors.

**Figure 5:**
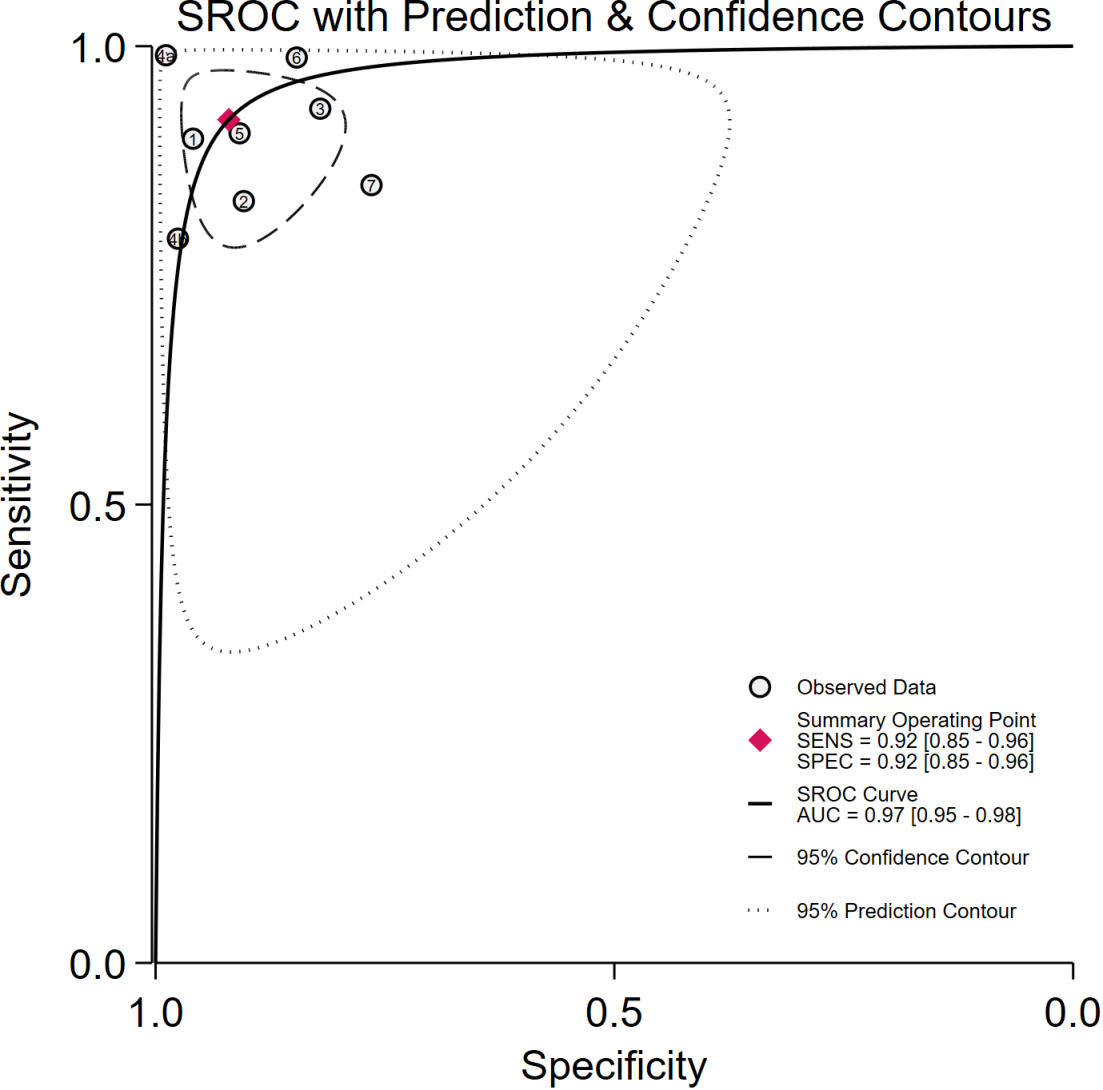
Summary receiver operating characteristic (SROC) curves for the diagnosis of pancreatic tumors using DL. Each circle indicates an individual study, red diamond represents summary sensitivity and specificity.

**Figure 6:**
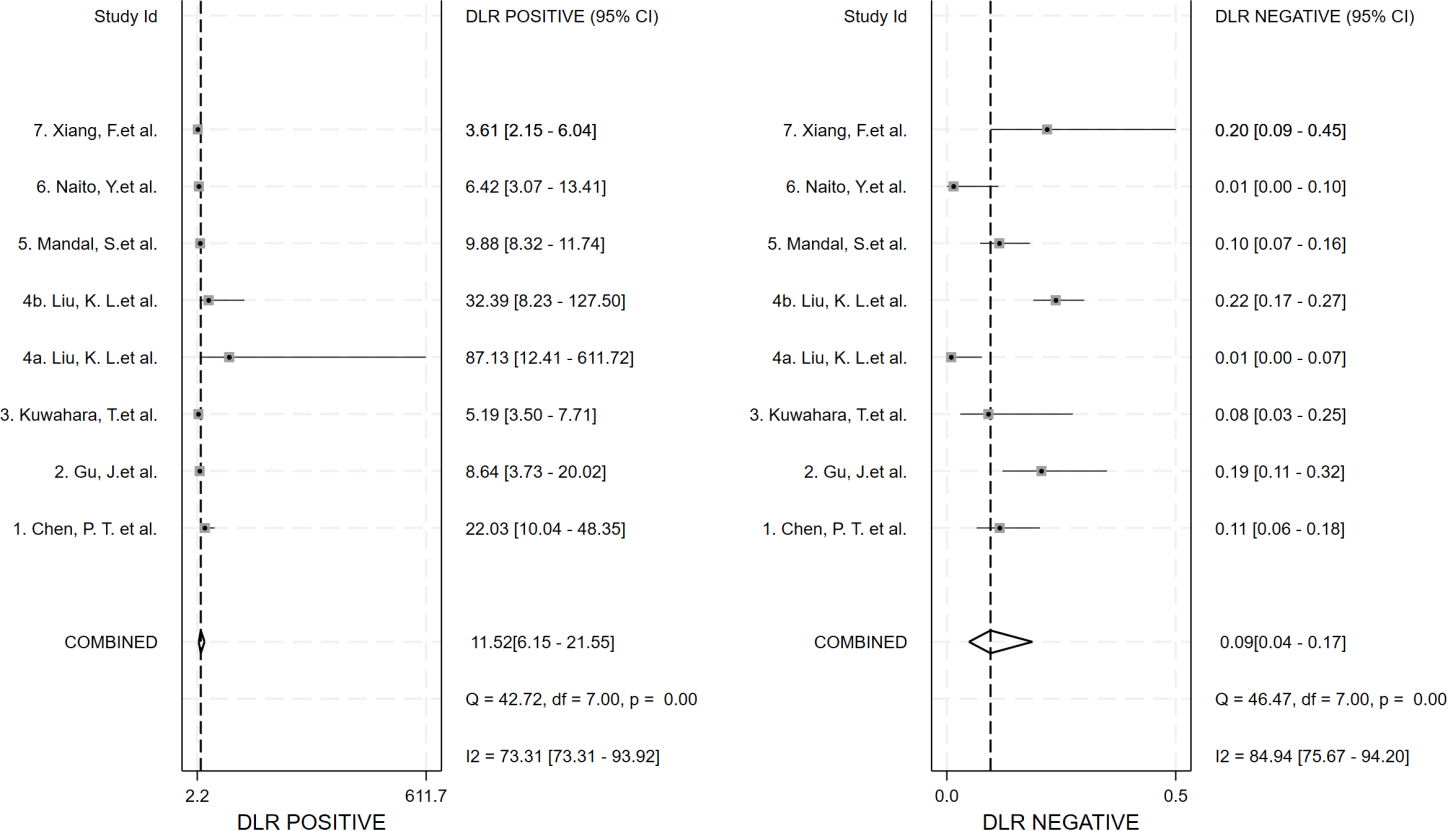
Forest plot for likelihood ratio after combination (LR+, LR-).

**Figure 7:**
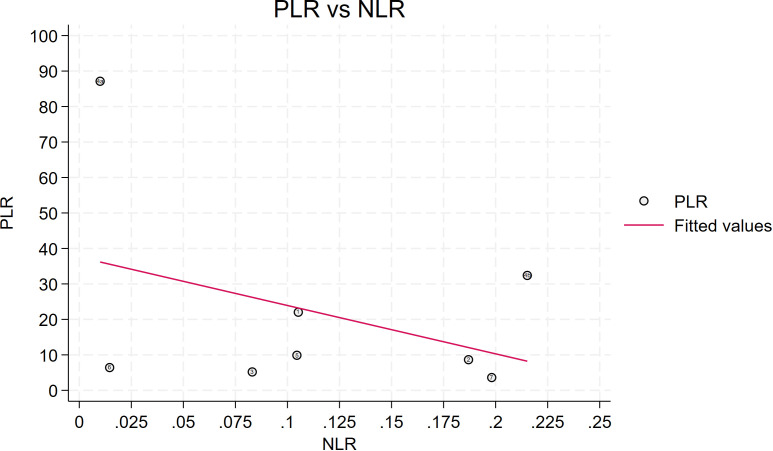
Likelihood ratio scatter plot is relatively scattered, and there may be large inter-study heterogeneity. PLR is the positive likelihood ratio and NLR is the negative likelihood ratio.

Seven studies provided enough data to construct 8 contingency tables, with a pooled sensitivity of 0.92 (95% CI: 0.85-0.96), specificity of 0.92 (95% CI: 0.85-0.96), and AUC of 0.97 (95% CI, 0.95.-0.98) (**Table 2**). The combined positive likelihood ratio and negative likelihood ratio were 11.52 (95% CI, 6.15-21.55) and 0.09 (95% CI, 0.04-0.17), respectively (**Figures 4**–**6**). The likelihood ratio scatter plot distribution is relatively scattered, and there may be large inter-study heterogeneity, which needs further analysis of the source of heterogeneity (**Figure 7**).

**Table 2:** Summary table of the results of the meta-analysis.

No.	Paper	Applications	TP	FP	FN	TN	Sensitivity	Specificity	AUC
1	Chen et al. (5)	CE-CT	98	6	11	141	0.90[0.83-0.95]	0.96[0.91-0.98]	0.96[0.94-0.99]
2	Gu et al. (3)	EUS	59	5	12	47	0.83[0.72-0.91]	0.90[0.79-0.97]	0.94[0.89-0.98]
3	Kuwahara, T.et al. (6)	EUS	41	21	3	96	0.93[0.81-0.99]	0.82[0.74-0.89]	0.90[0.84-0.97]
4a	Liu et al. (20)	CE-CT	100	1	1	87	0.99[0.95-1.00]	0.99[0.94-1.00]	1.00[1.00-1.00]
4b	Liu et al. (20)	CE-CT	222	2	59	80	0.79[0.74-0.84]	0.98[0.91-1.00]	0.92[0.89-0.95]
5	Mandal et al. (14)	CE-CT	162	128	17	1270	0.91[0.85-0.94]	0.91[0.89-0.92]	0.90[0.90-0.90]
6	Naito et al. (27)	EUS	80	6	1	33	0.99[0.93-1.00]	0.85[0.69-0.94]	0.98[0.96-1.00]
7	Xiang et al. (21)	CE-CT	28	12	5	39	0.85[0.68-0.95]	0.76[0.63-0.87]	0.86[0.77-0.94]
8	COMBINED						0.92[0.85-0.96]	0.92[0.85-0.96]	0.97[0.95-0.98]

Inter-study heterogeneity was quantified using the Mantel–Haenszel Q test and the I² statistic. Mantel-Haenszel Q test: The Q value is 43.72, the degrees of freedom are 7, and the p-value is 0.000 < 0.01, indicating significant heterogeneity among the studies. I² value: I² = 84.0% (95% CI 67.8 - 90.2%). Since the I² value is greater than 50%, it indicates that a significant proportion of the variation is caused by the heterogeneity among the studies.

Given that fewer than ten studies were available, we performed stratified analyses to identify potential sources of this heterogeneity. Studies were stratified by sample size (≤ 1–000 vs > 1 000), imaging modality (CE-CT vs EUS), deep-learning architecture (CNN vs non-CNN), and validation strategy (internal vs external).

In the CE-CT subgroup, heterogeneity remained pronounced (Q = 41.61, df = 4, p < 0.001; I² = 90.4%, 95% CI 79.9–94.2%). By contrast, the EUS subgroup exhibited negligible heterogeneity (Q = 1.53, df = 2, p = 0.464; I² = 0.0%, 95% CI 0.0–72.9%). The markedly lower heterogeneity in the EUS subset may reflect greater consistency in image acquisition, reader expertise, lesion size, or histopathological reference standards; however, the wide confidence interval (0.0–72.9%) indicates that imprecision due to the small number of studies cannot be excluded. None of the remaining stratifications materially reduced the observed heterogeneity (**Supplementary File 4**).

**Figure 8:**
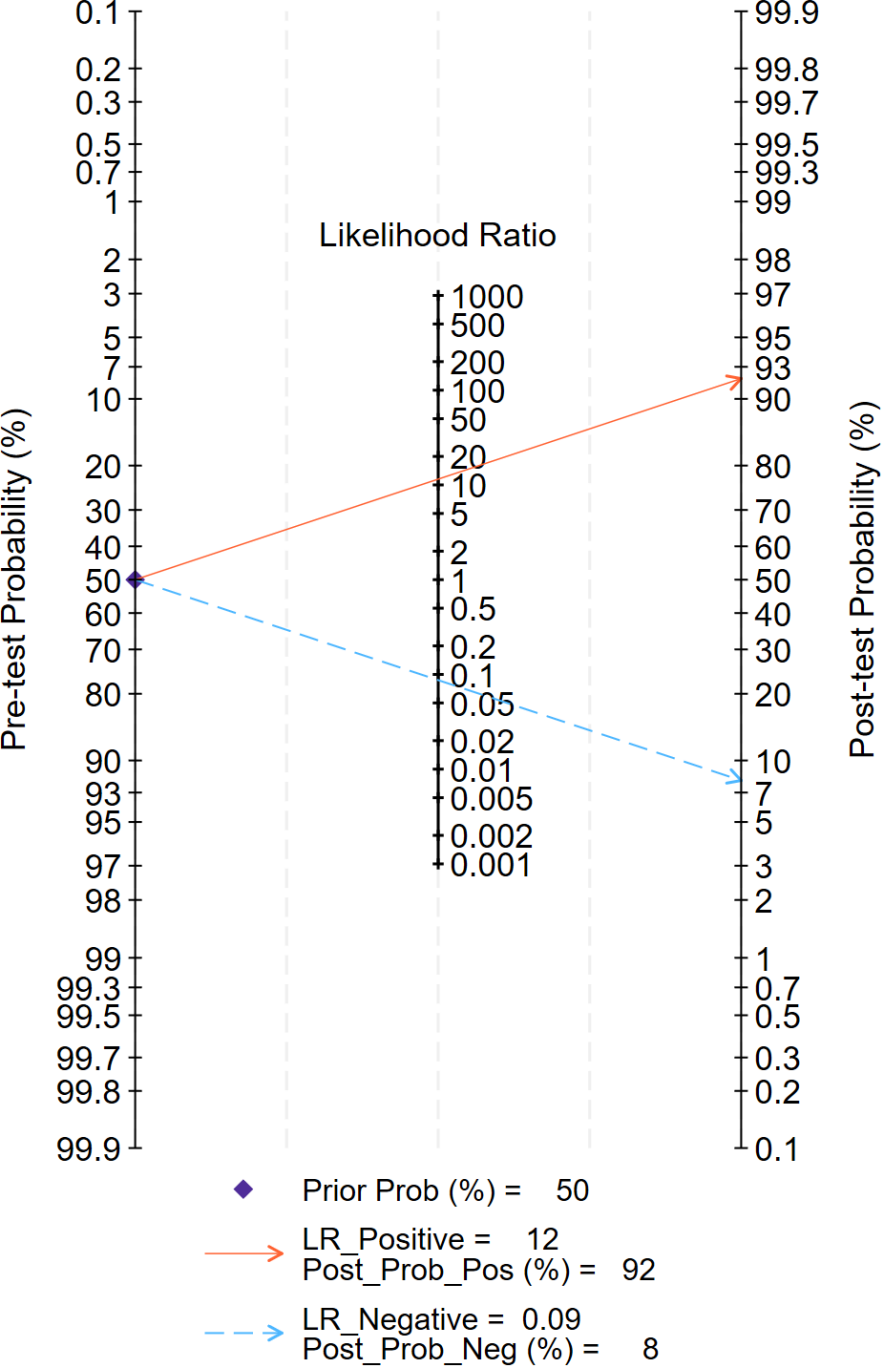
Fagan nomogram of the accuracy of DL in the diagnosis of pancreatic tumors.

The Fagan chart analysis, which assessed a 50% predicted probability to simulate a clinical situation, resulted in a 92% posterior probability of a positive test result, compared with a negative likelihood ratio of 0.09 and a negative posterior probability of 8% (**Figure 8**).

**Figure 9:**
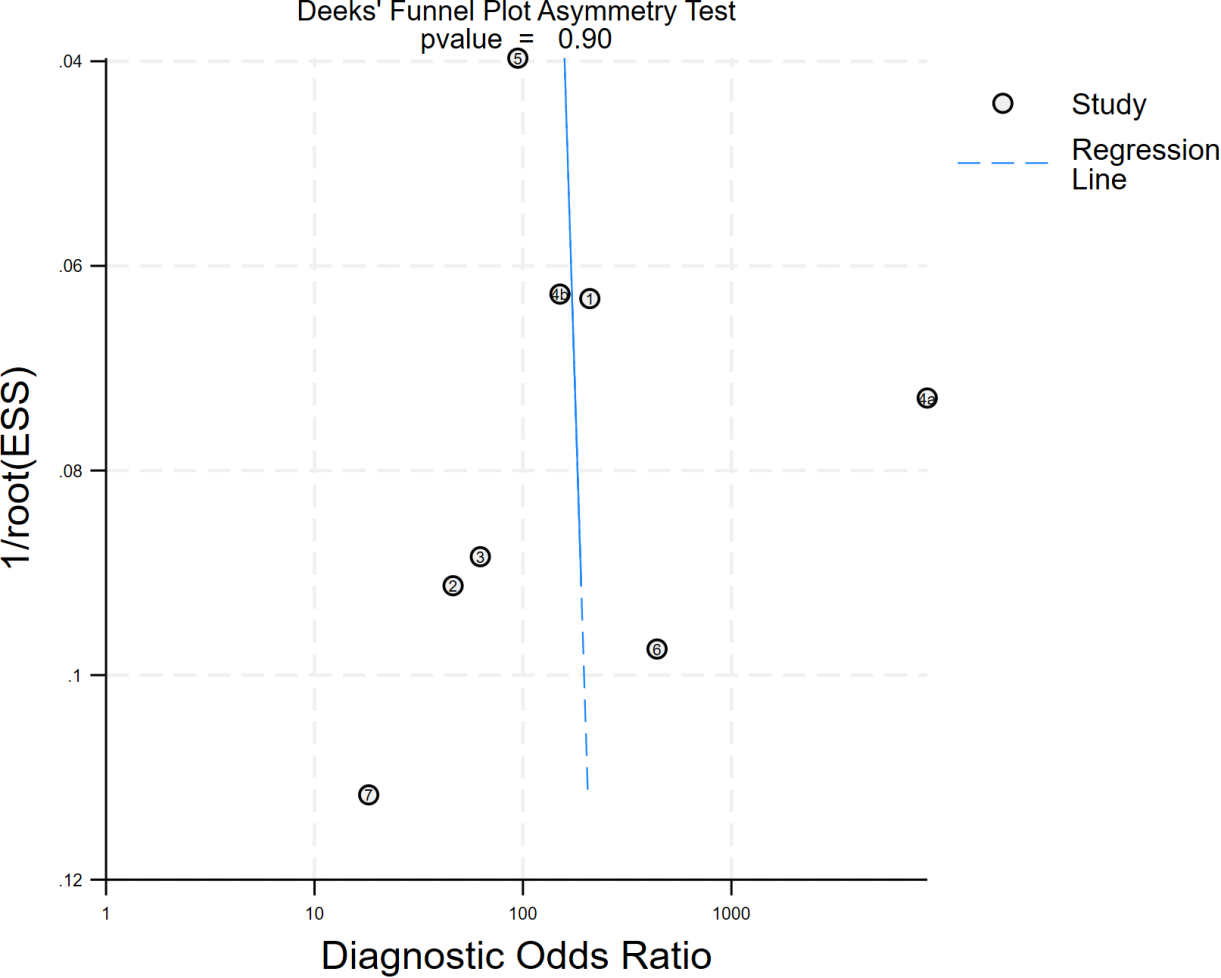
Deek funnel plot showing publication bias.

A Deeks funnel plot asymmetry test with a p value of 0.90 implies that the probability of obtaining the current or more extreme data, given the null hypothesis (i.e., no publication bias), is 90%. The funnel plot has good symmetry, and the distribution of the study results is in line with the expectation of random error, with no obvious signs of bias (**Figure 9**). The Egger regression yielded an intercept of 1.318 (p = 0.447; F-test p = 0.4465), indicating that the intercept did not significantly deviate from zero (**Supplementary File 5**). Consequently, there was no statistical evidence of small-study effects or publication bias.

## Discussion

We conducted a systematic review and meta-analysis on the research on deep learning for early diagnosis of pancreatic cancer over the past five years. Through the analysis of indicators such as accuracy, sensitivity, specificity, and AUC, we aimed to demonstrate the efficacy and clinical applicability of deep learning models in the early diagnosis of pancreatic cancer by learning from CE-CT, EUS images, as well as urine biomarkers, disease trajectories, etc. According to our meta-analysis, the accuracy rate of deep learning models for early diagnosis of pancreatic cancer has been at a relatively high level in the past five years, and the publication bias is relatively small. However, the heterogeneity among the studies is relatively strong. In this regard, we provided further explanations in the Limitations section.

Among the included studies, convolutional neural networks (CNN) were the most commonly used algorithm because it can easily handle multi-dimensional data by using a large number of parameters. At present, imaging indicators such as CE-CT and EUS seem to be more popular data for deep learning, but genomic data, RNA, urine biomarkers, and disease trajectories have also become new objects for deep learning, and perhaps they can become one of the main directions of future research for diagnosing and predicting pancreatic cancer using artificial intelligence algorithms.

In recent years, multi-modal approaches has also become a research hotspot, but its application in the early diagnosis of pancreatic cancer remains to be explored. Multi-modal approaches refer to the simultaneous generation of multiple omics data from the same sample, thereby providing a comprehensive view of biological system processes. Therefore, multi-omics data offer valuable and holistic insights into the complex interactions between biological mechanisms and biomarkers (46, 47). Multi-modal approaches have the potential to integrate different data sources (such as imaging data, genomics, and clinical records), thereby enriching the feature space and improving diagnostic accuracy (48). However, this integration is challenged by data heterogeneity, which arises from differences in data acquisition protocols, patient populations, and measurement scales. Optimization can be achieved from two perspectives. First, from the perspective of data sources, establishing standardized public databases can reduce heterogeneity caused by single data sources. Providing open-source code can help researchers better reproduce study results and reduce heterogeneity caused by different parameter settings. Second, from the perspective of model stability and generalizability, it is encouraged to conduct multicenter studies to train models with large amounts of data, thereby improving the stability and generalizability of models under different devices and operating habits (49, 50).

Deep learning algorithms typically contain a large number of parameters, which need to be learned through data to establish a complex mapping relationship between input features and output results. The more parameters, the stronger the model’s expressive power, but it is also prone to overfitting (i.e., performing well on the training set but having poor generalization ability on external validation sets) (51, 52). Large sample sizes can provide more diverse data distributions, helping deep learning models obtain high-robust features and improve generalization ability (53). Therefore, large sample size data is needed for model training. However, in our meta-analysis, only one study used more than 1000 samples, and other studies were limited by the size of the data. Therefore, in future research, multicenter large sample sizes or even multiple countries may become the main trend.

An interesting finding of the review is that the number of published studies on deep learning models for early diagnosis of pancreatic cancer has been on the rise since 2020. In our meta-analysis, the number of included studies has been on the rise in all years except for 2022 (**Figure 1**). The reason for the relatively small number of relevant studies in 2022 may be affected by the COVID-19 pandemic. Currently, deep learning for early diagnosis of pancreatic cancer is in a research boom, and this field requires more high-quality research.

Many studies describe deep learning models as “black boxes” models, and we cannot accurately explain their specific working principles (2, 6, 7, 45). Currently, there are studies in the medical field that have explained machine learning models, such as Castagno et al. using the KernelSHAP tool to evaluate the importance of features for model prediction and selecting the top 5 features as core variables to build a new model (54). Du et al. used the SHAP algorithm to explain the model for differentiating hepatocellular carcinoma using gray-scale ultrasound (55).

Furthermore, enhancing model interpretability is crucial for clinical acceptance, as it allows clinicians to understand and trust the decision-making processes of deep-learning algorithms (56). Techniques such as feature visualization, saliency maps, and model-agnostic interpretability methods can provide insights into how models derive their predictions, thereby bridging the gap between complex algorithms and clinical practice (57). As the complexity of models increases, such as deep learning models, the difficulty of model interpretability has significantly risen. However, to enhance the reliability of deep learning models and promote their clinical application in early diagnosis of pancreatic cancer, the interpretability of deep learning models may become the focus of research in the coming years (54, 55, 58).

### Limitations

Lv et al. observed that 10 included studies exhibited high heterogeneity; however, even after conducting subgroup analyses and sensitivity analyses, no source of heterogeneity was identified (18). In our Meta-analysis, we encountered the same predicament. Heterogeneity could not be reduced by subgroup analysis.

We analyzed this. Firstly, the number of included studies was limited. Although grouping could be done based on the above criteria, this might be one of the reasons for the high heterogeneity. Secondly, since the deep learning models currently applied in the studies are mostly modified versions of large models such as CNN, we cannot achieve complete uniformity in training the models, which might also be the main reason for the heterogeneity. Additionally, various parameter settings, image quality, and inspection equipment could be sources of heterogeneity, but further exploration is needed.

## Conclusion

We believe that this review will be helpful for the scientific community to better understand the application of deep learning technology in risk prediction and diagnosis of pancreatic cancer. Recent published studies have shown high accuracy rates. However, due to limitations in dataset size and diversity, insufficient model interpretability, difficulties in clinical validation and implementation, ethical and legal concerns, as well as the acceptance and trust of clinicians and patients, no deep learning models have been widely applied in clinical practice.

To promote the future clinical application of deep learning models, it is imperative to address several key issues: exploring multi-modal approaches, resolving data heterogeneity problems, enhancing the interpretability of DL models, designing large-sample prospective studies, and conducting external validations.

We also believe that there are still cutting-edge issues that need to be addressed in the risk prediction and diagnosis of pancreatic cancer using artificial intelligence technology, which are specifically reflected in the following four aspects: (1) Genomics, RNA, Urine biomarkers, Disease trajectories and other data seem to have also become new objects for deep learning, and perhaps can become one of the main directions for future research; (2) Multi-center large-sample or even multi-national samples may become the main theme; (3) Early diagnosis of pancreatic cancer smaller than 2 cm remains a challenge; (4) The interpretability of deep learning models and the integration of multi-modal approaches may become the focus of research in the coming years.

## Data Availability

The original contributions presented in the study are included in the article/**Supplementary Material**. Further inquiries can be directed to the corresponding author.
