# Supplementary material for: Diagnosis methods for pancreatic cancer with the technique of deep learning: a review and a meta-analysis
Source: Front Oncol. 2025 Aug 20;15:1597969. doi: 10.3389/fonc.2025.1597969 (PMC12404995; doi:10.3389/fonc.2025.1597969)
Supplement: Supplementary file 1 [file Supplementaryfile1.docx]

**Search strategy**

**Diagnosis methods for Pancreatic cancer with the technique of deep learning: a review and a meta-analysis**

**PUBMED**

**1. Type of disease: Pancreatic Neoplasm**

Neoplasm, Pancreatic

Pancreatic Neoplasm

Neoplasms, Pancreatic

Pancreas Neoplasms

Neoplasm, Pancreas

Neoplasms, Pancreas

Pancreas Neoplasm

Cancer of Pancreas

Pancreas Cancers

Cancer of the Pancreas

Pancreas Cancer

Cancer, Pancreas

Cancers, Pancreas

Cancers, Pancreas

Cancer, Pancreatic

Cancers, Pancreatic

Pancreatic Cancers

Pancreatic Carcinoma

Carcinoma, Pancreatic

Carcinomas, Pancreatic

Pancreatic Carcinomas

Pancreatic Acinar Carcinoma

Acinar Carcinoma, Pancreatic

Acinar Carcinomas, Pancreatic

Carcinoma, Pancreatic Acinar

Carcinomas, Pancreatic Acinar

Pancreatic Acinar Carcinomas

Search: **("Pancreatic Neoplasms"[Mesh]) OR (((((((((((((((((((((((((((Neoplasm, Pancreatic[Title/Abstract]) OR (Pancreatic Neoplasm[Title/Abstract])) OR (Neoplasms, Pancreatic[Title/Abstract])) OR (Pancreas Neoplasms[Title/Abstract])) OR (Neoplasm, Pancreas[Title/Abstract])) OR (Neoplasms, Pancreas[Title/Abstract])) OR (Pancreas Neoplasm[Title/Abstract])) OR (Cancer of Pancreas[Title/Abstract])) OR (Pancreas Cancers[Title/Abstract])) OR (Cancer of the Pancreas[Title/Abstract])) OR (Pancreas Cancer[Title/Abstract])) OR (Cancer, Pancreas[Title/Abstract])) OR (Cancers, Pancreas[Title/Abstract])) OR (Cancers, Pancreas[Title/Abstract])) OR (Cancer, Pancreatic[Title/Abstract])) OR (Cancers, Pancreatic[Title/Abstract])) OR (Pancreatic Cancers[Title/Abstract])) OR (Pancreatic Carcinoma[Title/Abstract])) OR (Carcinoma, Pancreatic[Title/Abstract])) OR (Carcinomas, Pancreatic[Title/Abstract])) OR (Pancreatic Carcinomas[Title/Abstract])) OR (Pancreatic Acinar Carcinoma[Title/Abstract])) OR (Acinar Carcinoma, Pancreatic[Title/Abstract])) OR (Acinar Carcinomas, Pancreatic[Title/Abstract])) OR (Carcinoma, Pancreatic Acinar[Title/Abstract])) OR (Carcinomas, Pancreatic Acinar[Title/Abstract])) OR (Pancreatic Acinar Carcinomas[Title/Abstract]))**

**2. Intervention: early Diagnosis**

Diagnoses

Diagnose

Diagnoses and Examinations

Diagnoses and Examination

Examination and Diagnoses

Examinations and Diagnoses

Antemortem Diagnosis

Antemortem Diagnoses

Diagnoses, Antemortem

Diagnosis, Antemortem

Postmortem Diagnosis

Diagnoses, Postmortem

Diagnosis, Postmortem

Postmortem Diagnoses

Search: **("Early Detection of Cancer"[Mesh]) OR (((((((((((Cancer Early Detection[Title/Abstract]) OR (Early Diagnosis of Cancer[Title/Abstract])) OR (Cancer Early Diagnosis[Title/Abstract])) OR (Cancer Screening[Title/Abstract])) OR (Screening, Cancer[Title/Abstract])) OR (Cancer Screening Tests[Title/Abstract])) OR (Cancer Screening Test[Title/Abstract])) OR (Screening Test, Cancer[Title/Abstract])) OR (Screening Tests, Cancer[Title/Abstract])) OR (Test, Cancer Screening[Title/Abstract])) OR (Tests, Cancer Screening[Title/Abstract]))**

**3. Research methods: deep learning**

Learning, Deep

Hierarchical Learning

Learning, Hierarchical

Search: **("Deep Learning"[Mesh]) OR (((Learning, Deep[Title/Abstract]) OR (Hierarchical Learning[Title/Abstract])) OR (Learning, Hierarchical[Title/Abstract]))**

**4. Time limit of literature: 2019.12.31-2024.12.31**

((("Pancreatic Neoplasms"[Mesh]) OR (((((((((((((((((((((((((((Neoplasm, Pancreatic[Title/Abstract]) OR (Pancreatic Neoplasm[Title/Abstract])) OR (Neoplasms, Pancreatic[Title/Abstract])) OR (Pancreas Neoplasms[Title/Abstract])) OR (Neoplasm, Pancreas[Title/Abstract])) OR (Neoplasms, Pancreas[Title/Abstract])) OR (Pancreas Neoplasm[Title/Abstract])) OR (Cancer of Pancreas[Title/Abstract])) OR (Pancreas Cancers[Title/Abstract])) OR (Cancer of the Pancreas[Title/Abstract])) OR (Pancreas Cancer[Title/Abstract])) OR (Cancer, Pancreas[Title/Abstract])) OR (Cancers, Pancreas[Title/Abstract])) OR (Cancers, Pancreas[Title/Abstract])) OR (Cancer, Pancreatic[Title/Abstract])) OR (Cancers, Pancreatic[Title/Abstract])) OR (Pancreatic Cancers[Title/Abstract])) OR (Pancreatic Carcinoma[Title/Abstract])) OR (Carcinoma, Pancreatic[Title/Abstract])) OR (Carcinomas, Pancreatic[Title/Abstract])) OR (Pancreatic Carcinomas[Title/Abstract])) OR (Pancreatic Acinar Carcinoma[Title/Abstract])) OR (Acinar Carcinoma, Pancreatic[Title/Abstract])) OR (Acinar Carcinomas, Pancreatic[Title/Abstract])) OR (Carcinoma, Pancreatic Acinar[Title/Abstract])) OR (Carcinomas, Pancreatic Acinar[Title/Abstract])) OR (Pancreatic Acinar Carcinomas[Title/Abstract]))) AND (("Deep Learning"[Mesh]) OR (((Learning, Deep[Title/Abstract]) OR (Hierarchical Learning[Title/Abstract])) OR (Learning, Hierarchical[Title/Abstract])))) AND (("Diagnosis"[Mesh]) OR ((((((((((((((Diagnoses[Title/Abstract]) OR (Diagnose[Title/Abstract])) OR (Diagnoses[Title/Abstract] AND Examinations[Title/Abstract])) OR (Diagnoses[Title/Abstract] AND Examination[Title/Abstract])) OR (Examination[Title/Abstract] AND Diagnoses[Title/Abstract])) OR (Examinations[Title/Abstract] AND Diagnoses[Title/Abstract])) OR (Antemortem Diagnosis[Title/Abstract])) OR (Antemortem Diagnoses[Title/Abstract])) OR (Diagnoses, Antemortem[Title/Abstract])) OR (Diagnosis, Antemortem[Title/Abstract])) OR (Postmortem Diagnosis[Title/Abstract])) OR (Diagnoses, Postmortem[Title/Abstract])) OR (Diagnosis, Postmortem[Title/Abstract])) OR (Postmortem Diagnoses[Title/Abstract]))) Filters applied: in the last 5 years.

**WOS**

(((((((((((((((((((((((((((AB=(Pancreatic Neoplasm)) OR AB=(Neoplasm, Pancreatic)) OR AB=(Pancreatic Neoplasm)) OR AB=(Neoplasms, Pancreatic)) OR AB=(Pancreas Neoplasms)) OR AB=(Neoplasm, Pancreas)) OR AB=(Neoplasms, Pancreas)) OR AB=(Pancreas Neoplasm)) OR AB=(Cancer of Pancreas)) OR AB=(Pancreas Cancers)) OR AB=(Cancer of the Pancreas)) OR AB=(Pancreas Cancer)) OR AB=(Cancer, Pancreas)) OR AB=(Cancers, Pancreas)) OR AB=(Cancers, Pancreas)) OR AB=(Cancer, Pancreatic)) OR AB=(Cancers, Pancreatic)) OR AB=(Pancreatic Cancers)) OR AB=(Pancreatic Carcinoma)) OR AB=(Carcinoma, Pancreatic)) OR AB=(Carcinomas, Pancreatic)) OR AB=(Pancreatic Carcinomas)) OR AB=(Pancreatic Acinar Carcinoma)) OR AB=(Acinar Carcinoma, Pancreatic)) OR AB=(Acinar Carcinomas, Pancreatic)) OR AB=(Carcinoma, Pancreatic Acinar)) OR AB=(Carcinomas, Pancreatic Acinar)) OR AB=(Pancreatic Acinar Carcinomas)

((((((((((((((AB=(Diagnosis)) OR AB=(Diagnoses)) OR AB=(Diagnose)) OR AB=(Diagnoses and Examinations)) OR AB=(Diagnoses and Examination)) OR AB=(Examination and Diagnoses)) OR AB=(Examinations and Diagnoses)) OR AB=(Antemortem Diagnosis)) OR AB=(Antemortem Diagnoses)) OR AB=(Diagnoses, Antemortem)) OR AB=(Diagnosis, Antemortem)) OR AB=(Postmortem Diagnosis)) OR AB=(Diagnoses, Postmortem)) OR AB=(Diagnosis, Postmortem)) OR AB=(Postmortem Diagnoses)

((((AB=(deep learning)) OR AB=(Learning, Deep)) OR AB=(Hierarchical Learning)) OR AB=(Learning, Hierarchical))

**IEEE**

((((((Abstract:deep learning) OR (Abstract:Learning, Deep) OR (Abstract:Hierarchical Learning) OR (Abstract:Learning, Hierarchical))) AND ((No Keywords Specified))) AND ((Abstract:Postmortem Diagnosis) OR (Abstract:Diagnoses, Postmortem) OR (Abstract:Diagnosis, Postmortem) OR (Abstract:Postmortem Diagnoses))) OR ((Abstract:Diagnosis) OR (Abstract:Diagnoses) OR (Abstract:Diagnose) OR (Abstract:Diagnoses and Examinations) OR (Abstract:Diagnoses and Examination) OR (Abstract:Examination and Diagnoses) OR (Abstract:Examinations and Diagnoses) OR (Abstract:Antemortem Diagnosis) OR (Abstract:Antemortem Diagnoses) OR (Abstract:Diagnoses, Antemortem) OR (Abstract:Diagnosis, Antemortem))) AND ((Document Title:Pancreatic Neoplasm) OR (Document Title:Pancreas Cancer) OR (Document Title:Cancer of Pancreas) OR (Document Title:Cancer of the Pancreas) OR (Document Title:Pancreatic Acinar Carcinoma) OR (Document Title:Pancreas Neoplasms) OR (Document Title:Neoplasm, Pancreatic) OR (Document Title:Neoplasms, Pancreatic) OR (Document Title:Pancreas Neoplasm) OR (Document Title:Cancer, Pancreas) OR (Document Title:Cancer, Pancreatic))

**Cochrane Library**

Search Name:

Date Run: 30/11/2024 18:38:55

Comment:

ID Search Hits

#1 MeSH descriptor: [Pancreatic Neoplasms] explode all trees 2872

#2 MeSH descriptor: [Diagnosis] explode all trees 477571

#3 MeSH descriptor: [Deep Learning] explode all trees 355
