# Supplementary material for: Diagnosis methods for pancreatic cancer with the technique of deep learning: a review and a meta-analysis
Source: Front Oncol. 2025 Aug 20;15:1597969. doi: 10.3389/fonc.2025.1597969 (PMC12404995; doi:10.3389/fonc.2025.1597969)
Supplement: Supplementary file 2 [file Supplementaryfile2.docx]

| **Table 1**. Publications reporting on deep learning use in early diagnosis of pancreatic cancer. | | | | | | | | | | |  |  |
| --- | --- | --- | --- | --- | --- | --- | --- | --- | --- | --- | --- | --- |
| Paper | Year | Applications | Model | Sample | | | Performance scores | | | | | |
|  |  |  |  | Total | PDAC | Non-PDAC | Sensitivity\Recall | Specificity | Accuracy | AUC | Precision | F1-score |
| Chen et al.[1] | 2023 | CE-CT | CNN | 256 | 109 | 147 | 0.899 | 0.959 | 93.36% | 0.96 | 0.9423 | 0.9198 |
| Xiang et al.[2] | 2024 | CE-CT | ResNet50 network | 84 | 33 | 51 | 0.85 | 0.76 | 80% | 0.857 | 0.7 | 0.767 |
| Liu et al.[3] | 2020 | CE-CT | CNN | 189 | 101 | 88 | 0.99 | 0.989 | 98.90% | 0.999 | 0.9901 | 0.9902 |
|  |  |  |  | 363 | 281 | 82 | 0.79 | 0.976 | 83.20% | 0.92 | 0.895 | 0.838 |
| Mandal et al.[4] | 2024 | CE-CT | nnU-Net + MIL +C NN | 1577 | 179 | 1398 | 0.905 | 0.908 | 90.70% | 0.903 | 0.5586 | 0.6908 |
| Naito et al.[5] | 2021 | EUS | CNN | 120 | 81 | 39 | 0.9302 | 0.9706 | 94.17% | 0.9836 | 0.9302 | 0.9566 |
| Gu et al.[6] | 2024 | EUS | deep‐learning radiomics(DLR) | 123 | 71 | 52 | 0.831 | 0.904 | 86.18% | 0.936 | 0.9221 | 0.874 |
| Hu et al.[7] | 2024 | EUS | Modified Faster R-CNN (M-F-RCNN) | 1000 | 585 | 415 | 0.917 | 0.915 | 91.60% | 0.916 | 0.939 | 0.916 |
| Kuwahara, T.et al.[8] | 2023 | EUS | Deep convolutional generative adversarial network (DCGAN) | 161 | 117 | 44 | 0.94 | 0.822 | 90.70% | 0.901 | 0.932 | 0.936 |
| Tang et al.[9] | 2023 | Contrast-enhanced harmonic endoscopic ultrasound (CH-EUS) | CH-EUS MASTER | 39 | 26 | 13 | 0.909 | 100% | 93.8% | 0.955 | 0.96 | 94.6% |
| Hong et al.[10] | 2024 | Pathological examination | effentnet -b3 | 37 | 37 | - | 0.985 | - | 97.06% | 0.998 | 0.992 | 0.988 |
| Fu et al.[11] | 2021 | Pathological examination | CNN model | 9948 | 4960 | 4988 | 0.9736 | 0.9335 | 95.91% | 0.993 | 0.9362 | 0.9546 |
| Placido et al[12]. | 2023 | Disease trajectories | DNPR model | 9,000,000 | 27,900 | 8,972,100 | - | - | - | 0.88 | - | - |
| Park et al. [13] | 2022 | Disease trajectories | “grouped” neural network (GrpNN)model | 30,195 | 7,124 | - | - | - | - | 0.671 | - | - |
| Acer et al.[14] | 2023 | Urine biomarkers | GBC | 590 | 199 | 391 | 0.9245 | 0.9333 | 92.99% | 0.9761 | 0.9368 | 0.9306 |
| Karar et al.[15] | 2023 | Urine biomarkers | 1D CNN-LSTM | 590 | 199 | 391 | 1.00 | 0.97 | 97% | 0.98 | 0.96 | 0.98 |
| Hong et al.[16] | 2024 | RNA | HATZFS | - | - | - | - | - | - | - | - | - |
| Darmofal et al.[17] | 2024 | DNA | Genome-Derived-Diagnosis Ensemble (GDD-ENS) | - | - | - | - | - | 93% | - | - | - |

1. Chen, P.T., et al., *Pancreatic Cancer Detection on CT Scans with Deep Learning: A Nationwide Population-based Study.* Radiology, 2023. **306**(1): p. 172-182.

2. Xiang, F., et al., *A deep learning model based on contrast-enhanced computed tomography for differential diagnosis of gallbladder carcinoma.* Hepatobiliary and Pancreatic Diseases International, 2024. **23**(4): p. 376-384.

3. Liu, K.L., et al., *Deep learning to distinguish pancreatic cancer tissue from non-cancerous pancreatic tissue: a retrospective study with cross-racial external validation.* Lancet Digit Health, 2020. **2**(6): p. e303-e313.

4. Mandal, S., et al., *Weakly supervised large-scale pancreatic cancer detection using multi-instance learning.* Frontiers in Oncology, 2024. **14**.

5. Naito, Y., et al., *A deep learning model to detect pancreatic ductal adenocarcinoma on endoscopic ultrasound-guided fine-needle biopsy.* Sci Rep, 2021. **11**(1): p. 8454.

6. Gu, J., et al., *Prospective assessment of pancreatic ductal adenocarcinoma diagnosis from endoscopic ultrasonography images with the assistance of deep learning.* Cancer, 2023. **129**(14): p. 2214-2223.

7. Hu, S.S., et al., *Enhancing physician support in pancreatic cancer diagnosis: New M-F-RCNN artificial intelligence model using endoscopic ultrasound.* Endoscopy International Open, 2024. **12**(11): p. E1277-E1284.

8. Kuwahara, T., et al., *Artificial intelligence using deep learning analysis of endoscopic ultrasonography images for the differential diagnosis of pancreatic masses.* Endoscopy, 2023. **55**(2): p. 140-149.

9. Tang, A.L., et al., *Contrast-enhanced harmonic endoscopic ultrasound (CH-EUS) MASTER: A novel deep learning-based system in pancreatic mass diagnosis.* Cancer Medicine, 2023. **12**(7): p. 7962-7973.

10. Hong, S.J., et al., *Convolutional neural network model for automatic recognition and classification of pancreatic cancer cell based on analysis of lipid droplet on unlabeled sample by 3D optical diffraction tomography.* Computer Methods and Programs in Biomedicine, 2024. **246**.

11. Fu, H., et al., *Automatic Pancreatic Ductal Adenocarcinoma Detection in Whole Slide Images Using Deep Convolutional Neural Networks.* Frontiers in Oncology, 2021. **11**.

12. Placido, D., et al., *A deep learning algorithm to predict risk of pancreatic cancer from disease trajectories.* Nature Medicine, 2023. **29**(5): p. 1113-+.

13. Park, J., et al., *Deep learning on time series laboratory test results from electronic health records for early detection of pancreatic cancer.* J Biomed Inform, 2022. **131**: p. 104095.

14. Acer, I., et al., *Early diagnosis of pancreatic cancer by machine learning methods using urine biomarker combinations.* Turkish Journal of Electrical Engineering and Computer Sciences, 2023. **31**(1): p. 112-125.

15. Karar, M.E., N. El-Fishawy, and M. Radad, *Automated classification of urine biomarkers to diagnose pancreatic cancer using 1-D convolutional neural networks.* Journal of Biological Engineering, 2023. **17**(1).

16. Hong, J., et al., *HATZFS predicts pancreatic cancer driver biomarkers by hierarchical reinforcement learning and zero-forcing set.* Expert Systems with Applications, 2025. **260**.

17. Darmofal, M., et al., *Deep-Learning Model for Tumor-Type Prediction Using Targeted Clinical Genomic Sequencing Data.* Cancer Discovery, 2024. **14**(6): p. 1064-1081.
