# Supplementary material for: Diagnosis methods for pancreatic cancer with the technique of deep learning: a review and a meta-analysis
Source: Front Oncol. 2025 Aug 20;15:1597969. doi: 10.3389/fonc.2025.1597969 (PMC12404995; doi:10.3389/fonc.2025.1597969)
Supplement: Supplementary file 4 [file Supplementaryfile4.docx]

**1.Heterogeneity test**

metan tp fn fp tn, rr fixed nograph

Studies included: 8

Participants included: 2873

Meta-analysis pooling of Risk Ratios

using the Mantel-Haenszel method

--------------------------------------------------------------------

| Risk

Study | ratio [95% Conf. Interval] % Weight

---------------------+----------------------------------------------

1 | 22.028 10.035 48.350 6.99

2 | 8.642 3.731 20.017 7.90

3 | 5.192 3.495 7.711 15.70

4 | 87.129 12.410 611.723 1.46

5 | 32.391 8.229 127.500 4.24

6 | 9.885 8.324 11.737 39.74

7 | 6.420 3.074 13.408 11.08

8 | 3.606 2.154 6.037 12.90

---------------------+----------------------------------------------

Overall, MH | 10.787 8.878 13.106 100.00

--------------------------------------------------------------------

Test of overall effect = 1: z = 23.938 p = 0.000

Heterogeneity measures, calculated from the data

with Conf. Intervals based on non-central chi² (common-effect) distribution for Q

---------------------------------------------------------

Measure | Value df p-value

---------------------+-----------------------------------

Mantel-Haenszel Q | 43.72 7 0.000

| -[95% Conf. Interval]-

H | 2.499 1.764 3.189

I² (%) | 84.0% 67.8% 90.2%

---------------------------------------------------------

H = relative excess in Mantel-Haenszel Q over its degrees-of-freedom

I² = proportion of total variation in effect estimate due to between-study heterogeneity (based on Q)

.

**2. Subgroup analysis**

Sample size (≤1000)

metan tp fn fp tn, rr fixed nograph

Studies included: 7

Participants included: 1296

Meta-analysis pooling of Risk Ratios

using the Mantel-Haenszel method

--------------------------------------------------------------------

| Risk

Study | ratio [95% Conf. Interval] % Weight

---------------------+----------------------------------------------

1 | 22.028 10.035 48.350 11.60

2 | 8.642 3.731 20.017 13.10

3 | 5.192 3.495 7.711 26.06

4 | 87.129 12.410 611.723 2.43

5 | 32.391 8.229 127.500 7.03

6 | 6.420 3.074 13.408 18.39

7 | 3.606 2.154 6.037 21.40

---------------------+----------------------------------------------

Overall, MH | 11.383 8.463 15.309 100.00

--------------------------------------------------------------------

Test of overall effect = 1: z = 16.084 p = 0.000

Heterogeneity measures, calculated from the data

with Conf. Intervals based on non-central chi² (common-effect) distribution for Q

---------------------------------------------------------

Measure | Value df p-value

---------------------+-----------------------------------

Mantel-Haenszel Q | 46.11 6 0.000

| -[95% Conf. Interval]-

H | 2.772 1.971 3.524

I² (%) | 87.0% 74.2% 91.9%

---------------------------------------------------------

H = relative excess in Mantel-Haenszel Q over its degrees-of-freedom

I² = proportion of total variation in effect estimate due to between-study heterogeneity (based on Q)

Sample size ( >1000)

Studies included: 1

Participants included: 1577

Meta-analysis pooling of Risk Ratios

using the Mantel-Haenszel method

--------------------------------------------------------------------

| Risk

Study | ratio [95% Conf. Interval] % Weight

---------------------+----------------------------------------------

1 | 9.885 8.324 11.737 100.00

---------------------+----------------------------------------------

Overall, MH | 9.885 8.324 11.737 100.00

--------------------------------------------------------------------

Test of overall effect = 1: z = 26.136 p = 0.000

.

Diagnostic method (CE-CT)

metan tp fn fp tn, rr fixed nograph

Studies included: 5

Participants included: 2469

Meta-analysis pooling of Risk Ratios

using the Mantel-Haenszel method

--------------------------------------------------------------------

| Risk

Study | ratio [95% Conf. Interval] % Weight

---------------------+----------------------------------------------

1 | 22.028 10.035 48.350 10.70

2 | 87.129 12.410 611.723 2.24

3 | 32.391 8.229 127.500 6.48

4 | 9.885 8.324 11.737 60.84

5 | 3.606 2.154 6.037 19.74

---------------------+----------------------------------------------

Overall, MH | 13.132 10.386 16.603 100.00

--------------------------------------------------------------------

Test of overall effect = 1: z = 21.517 p = 0.000

Heterogeneity measures, calculated from the data

with Conf. Intervals based on non-central chi² (common-effect) distribution for Q

---------------------------------------------------------

Measure | Value df p-value

---------------------+-----------------------------------

Mantel-Haenszel Q | 41.61 4 0.000

| -[95% Conf. Interval]-

H | 3.225 2.230 4.152

I² (%) | 90.4% 79.9% 94.2%

---------------------------------------------------------

H = relative excess in Mantel-Haenszel Q over its degrees-of-freedom

I² = proportion of total variation in effect estimate due to between-study heterogeneity (based on Q)

Diagnostic method (EUS)

metan tp fn fp tn, rr fixed nograph

Studies included: 3

Participants included: 404

Meta-analysis pooling of Risk Ratios

using the Mantel-Haenszel method

--------------------------------------------------------------------

| Risk

Study | ratio [95% Conf. Interval] % Weight

---------------------+----------------------------------------------

1 | 8.642 3.731 20.017 22.77

2 | 5.192 3.495 7.711 45.28

3 | 6.420 3.074 13.408 31.95

---------------------+----------------------------------------------

Overall, MH | 6.370 4.432 9.155 100.00

--------------------------------------------------------------------

Test of overall effect = 1: z = 10.005 p = 0.000

Heterogeneity measures, calculated from the data

with Conf. Intervals based on non-central chi² (common-effect) distribution for Q

---------------------------------------------------------

Measure | Value df p-value

---------------------+-----------------------------------

Mantel-Haenszel Q | 1.53 2 0.464

| -[95% Conf. Interval]-

H | 0.876 1.000 1.921

I² (%) | 0.0% 0.0% 72.9%

---------------------------------------------------------

H = relative excess in Mantel-Haenszel Q over its degrees-of-freedom

I² = proportion of total variation in effect estimate due to between-study heterogeneity (based on Q)

Deep learning model (CNN)

metan tp fn fp tn, rr fixed nograph

Studies included: 4

Participants included: 928

Meta-analysis pooling of Risk Ratios

using the Mantel-Haenszel method

--------------------------------------------------------------------

| Risk

Study | ratio [95% Conf. Interval] % Weight

---------------------+----------------------------------------------

1 | 22.028 10.035 48.350 29.41

2 | 87.129 12.410 611.723 6.15

3 | 32.391 8.229 127.500 17.82

4 | 6.420 3.074 13.408 46.62

---------------------+----------------------------------------------

Overall, MH | 20.603 12.516 33.914 100.00

--------------------------------------------------------------------

Test of overall effect = 1: z = 11.897 p = 0.000

Heterogeneity measures, calculated from the data

with Conf. Intervals based on non-central chi² (common-effect) distribution for Q

---------------------------------------------------------

Measure | Value df p-value

---------------------+-----------------------------------

Mantel-Haenszel Q | 12.18 3 0.007

| -[95% Conf. Interval]-

H | 2.015 1.000 3.025

I² (%) | 75.4% 0.0% 89.1%

---------------------------------------------------------

H = relative excess in Mantel-Haenszel Q over its degrees-of-freedom

I² = proportion of total variation in effect estimate due to between-study heterogeneity (based on Q)

Deep learning model (non-CNN)

metan tp fn fp tn, rr fixed nograph

Studies included: 4

Participants included: 1945

Meta-analysis pooling of Risk Ratios

using the Mantel-Haenszel method

--------------------------------------------------------------------

| Risk

Study | ratio [95% Conf. Interval] % Weight

---------------------+----------------------------------------------

1 | 8.642 3.731 20.017 10.36

2 | 5.192 3.495 7.711 20.59

3 | 9.885 8.324 11.737 52.13

4 | 3.606 2.154 6.037 16.92

---------------------+----------------------------------------------

Overall, MH | 7.727 6.562 9.100 100.00

--------------------------------------------------------------------

Test of overall effect = 1: z = 24.513 p = 0.000

Heterogeneity measures, calculated from the data

with Conf. Intervals based on non-central chi² (common-effect) distribution for Q

---------------------------------------------------------

Measure | Value df p-value

---------------------+-----------------------------------

Mantel-Haenszel Q | 20.24 3 0.000

| -[95% Conf. Interval]-

H | 2.598 1.446 3.642

I² (%) | 85.2% 52.2% 92.5%

---------------------------------------------------------

H = relative excess in Mantel-Haenszel Q over its degrees-of-freedom

I² = proportion of total variation in effect estimate due to between-study heterogeneity (based on Q)

Validation type (Internal validation)

metan tp fn fp tn, rr fixed nograph

Studies included: 6

Participants included: 2387

Meta-analysis pooling of Risk Ratios

using the Mantel-Haenszel method

--------------------------------------------------------------------

| Risk

Study | ratio [95% Conf. Interval] % Weight

---------------------+----------------------------------------------

1 | 22.028 10.035 48.350 7.95

2 | 5.192 3.495 7.711 17.87

3 | 87.129 12.410 611.723 1.66

4 | 9.885 8.324 11.737 45.23

5 | 6.420 3.074 13.408 12.61

6 | 3.606 2.154 6.037 14.68

---------------------+----------------------------------------------

Overall, MH | 9.939 8.262 11.956 100.00

--------------------------------------------------------------------

Test of overall effect = 1: z = 24.359 p = 0.000

Heterogeneity measures, calculated from the data

with Conf. Intervals based on non-central chi² (common-effect) distribution for Q

---------------------------------------------------------

Measure | Value df p-value

---------------------+-----------------------------------

Mantel-Haenszel Q | 35.28 5 0.000

| -[95% Conf. Interval]-

H | 2.656 1.776 3.474

I² (%) | 85.8% 68.3% 91.7%

---------------------------------------------------------

H = relative excess in Mantel-Haenszel Q over its degrees-of-freedom

I² = proportion of total variation in effect estimate due to between-study heterogeneity (based on Q)

Validation type (External validation)

metan tp fn fp tn, rr fixed nograph

Studies included: 2

Participants included: 486

Meta-analysis pooling of Risk Ratios

using the Mantel-Haenszel method

--------------------------------------------------------------------

| Risk

Study | ratio [95% Conf. Interval] % Weight

---------------------+----------------------------------------------

1 | 8.642 3.731 20.017 65.09

2 | 32.391 8.229 127.500 34.91

---------------------+----------------------------------------------

Overall, MH | 16.934 7.854 36.511 100.00

--------------------------------------------------------------------

Test of overall effect = 1: z = 7.218 p = 0.000

Heterogeneity measures, calculated from the data

with Conf. Intervals based on non-central chi² (common-effect) distribution for Q

---------------------------------------------------------

Measure | Value df p-value

---------------------+-----------------------------------

Mantel-Haenszel Q | 3.32 1 0.068

| -[95% Conf. Interval]-

H | 1.823 1.000 3.485

I² (%) | 69.9% 0.0% 91.8%

---------------------------------------------------------

H = relative excess in Mantel-Haenszel Q over its degrees-of-freedom

I² = proportion of total variation in effect estimate due to between-study heterogeneity (based on Q)
