# Supplementary material for: Diagnosis methods for pancreatic cancer with the technique of deep learning: a review and a meta-analysis
Source: Front Oncol. 2025 Aug 20;15:1597969. doi: 10.3389/fonc.2025.1597969 (PMC12404995; doi:10.3389/fonc.2025.1597969)
Supplement: Supplementary file 5 [file Supplementaryfile5.docx]

Egger regression

.

. regress std_eff inv_se, vce(robust)

Linear regression Number of obs = 8

F(1, 6) = 27.55

Prob > F = 0.0019

R-squared = 0.7672

Root MSE = 1.9838

------------------------------------------------------------------------------

| Robust

std_eff | Coefficient std. err. t P>|t| [95% conf. interval]

-------------+----------------------------------------------------------------

inv_se | 3.843722 .7322523 5.25 0.002 2.051965 5.635478

_cons | 1.317611 1.617805 0.81 0.447 -2.641015 5.276236

------------------------------------------------------------------------------

.

. * Significant non-zero constant term indicates a possible small sample effect / publication bias

.

. test _cons = 0

( 1) _cons = 0

F( 1, 6) = 0.66

Prob > F = 0.4465

.
